# Mammoth and Elephant Phylogenetic Relationships: *Mammut Americanum*, the Missing Outgroup

**Published:** 2007-03-29

**Authors:** Ludovic Orlando, Catherine Hänni, Christophe J. Douady

**Affiliations:** 1 Paleogenetics and Molecular Evolution; IFR 128, Lyon, F-69007, France; Université Lyon 1, Lyon, F-69007, France; CNRS UMR 5242, INRA, Institut de Génomique Fonctionnelle de Lyon, Ecole Normale Supérieure de Lyon, 46 Allée d’Italie, Lyon, F-69364 Cédex 07, France.; 2 CNRS UMR 5023, Laboratoire d’Ecologie des Hydrosystèmes Fluviaux, Université Claude Bernard Lyon 1, 6 rue R. Dubois, Bat. Darwin-C, F-69622 Villeurbanne Cédex, France.

**Keywords:** Phylogeny, Ancient DNA, Elephantidae, Mammoth

## Abstract

At the morphological level, the woolly mammoth has most often been considered as the sister-species of Asian elephants, but at the DNA level, different studies have found support for proximity with African elephants. Recent reports have increased the available sequence data and apparently solved the discrepancy, finding mammoths to be most closely related to Asian elephants. However, we demonstrate here that the three competing topologies have similar likelihood, bayesian and parsimony supports. The analysis further suggests the inadequacy of using Sirenia or Hyracoidea as outgroups. We therefore argue that orthologous sequences from the extinct American mastodon will be required to definitively solve this long-standing question.

## Introduction

The mammoth lineage offers one of the most complete palaeontological records among vertebrates (Lister and Sher, 2001). Large sampling and dating evidence have contributed to set morphological adaptive changes in a precise geographical and temporal framework (Lister et al. 2005). The elephant and mammoth lineages probably diverged some 4–6 million years ago (MYA) in Africa (Shoshani and Tassy, 2005; Todd, 2006), but it is around 3 MYA that mammoths spread across the temperate and wooded habitats from Europe to China (Lister et al. 2005). Populations from China and Northern Siberia, adapted to cold and steppe conditions, progressively supplanted older forms (Lister and Sher, 2001). By 200 KYA, the woolly mammoth stage (*Mammuthus primigenius*) was reached in Northern Siberia and started to spread westwards to Europe. It spread later eastwards across the Beringia into Northern America, where descendants of ancestral forms adapted to the temperate grasslands already lived, *Mammuthus columbi* as well as pigmy mammoths (*Mammuthus exilis*) (Agenbroad, 2005). The cooling from the end of the Last Ice Age considerably restricted their habitat and precipitated their extinction; at the beginning of the Holocene, mammoths only survived in small refugial islands from the Arctic and Bering Sea and by 3.7 KYA the very last specimen disappeared (Vartanyan et al. 1993; Guthrie et al. 2004; Stuart et al. 2004).

Several points in this impressively well documented model are still debated though. Among these, the tempo and mode for mammoth extinction, especially with regards to possible overkilling by hunters, is perhaps the most controversial issue (Agenbroad, 2005; Stuart, 2005). But the question of the origin of the mammoth lineage has also received much attention in the last decade. Palaeontologists have long found support in morphological characters for a sister group relationship between mammoths and Asian elephants (*Elephas*), rather than African elephants (*Loxodonta*) (Maglio, 1973). This model has received additional support from the analysis of new characters, such as the hyoid apparatus (an association of nine bones connected to the cranium, the tongue and the larynx) (Shoshani and Marchant, 2001). Surprisingly, the very first mammoth DNA sequence exhibited minimum genetic distance with extant African but not Asian elephants (Hagelberg et al. 1994; Table 1). Some larger sequence datasets grouped mammoth and *Elephas* (Yang et al. 1996; Ozawa et al. 1997; Table 1). But reanalysis of these data found again a grouping of *Mammuthus* and *Loxodonta*. These results called into question (i) the validity of morphological synapomorphies between mammoths and Asian elephants (Noro et al. 1998; Thomas et al. 2000; Debruyne et al. 2003), and (ii) the authenticity of some of the previously reported sequences (Yang et al. 1996; Thomas et al. 2000; Debruyne et al. 2003). Most recently, partial nuclear gene sequences (Capelli et al. 2006) and complete mitochondrial genomes (Krause et al. 2006; Rogaev et al. 2006) have revived the debate, showing support for the (*Mammuthus, Elephas*) clade. Despite some claims to the contrary, *Mammuthus* affinities are still far from being conclusively settled, with different topologies supported by Rogaev et al. (2006), Krause et al. (2006), and Capelli et al. (2006; summarized in Table 1). In this study, we evaluate for the first time the phylogenetic signal contained in all the data, by combining all the available nuclear and mitochondrial genes.

## Material and Methods

### Data construction

Mitochondrial sequences were retrieved from Genbank and manually aligned using the Seaview software (Galtier et al. 1996). An alignment of the nuclear sequences available for elephantids was kindly provided by A.D. Greenwood. The complete data sets as a whole and 16 different partitions were further analyzed (Table 2).

### Most likely topologies and number of synapomorphies

For all data sets and partition subsets we estimated the most likely topologies in favor of (*Mammuthus*, *Elephas*), (*Mammuth, Loxodonta*) and (*Elephas, Loxodonta*) clustering. All computations were done using PAUP (Swofford, 2002) and the best fitted model according to Akaike criterion (Akaike, 1974) as implemented in Modeltest (Posada and Crandall, 1998). Approximatively Unbiased (Shimodaira, 2002), Kishino-Hasegawa (1989) and Shimodaira-Hasegawa (1999) tests were done using CONSEL (Shimodaira and Hasegawa, 2001). The number of synapomorphies for each of these alternatives were all collected via direct pairwise comparisons and possible significant differences were evaluated using a Chi-square test.

### Partitioned bayesian inferences and partition contents

In order to test for phylogenetic support in a partitioned Bayesian framework we analyzed two partition schemes using MRBAYES v3.1.2. Both analyses employed a GTR model of evolution assuming a fraction of invariant sites and a rate heterogeneity across sites. For each, two sets of four chains sampled every 100 generations were ran until the average standard deviation of split frequencies between the two set fell below the default critical value of 0.01 using a burn-in fraction of 25%. To ensure that consensus trees were based on a rather large collection of trees, average standard deviation of split frequencies were only evaluated every 100000 generations. Our first partition scheme was designed to ensure that sites under different evolutionary dynamic received independent evaluation. Thus we defined 10 partitions after one for each nuclear codon position, one for each mitochondrial codon positions, one for mitochondrial ribosomal RNA positions, one for mitochondrial transfer RNA positions, one for nuclear non coding positions and one for mitochondrial non coding positions. Our second scheme allowed independent estimation of model parameters for each gene or gene fragment. Mitochondrial non coding positions (to the exception of the D-loop positions) were all lump together, leading to a total of 46 partitions). These schemes are referred as P10 and P46 respectively.

Finally the information content of the different genes were estimated using the hidden branch support approach described in Gatesy et al. (1999). These estimations were done both in a parsimony and likelihood framework using PAUP (Swofford, 2002).

## Results/Discussion

We first started by computing the likelihood of the three alternative topologies under the best-fitting model of molecular evolution using different sets of sequences (Table 2: protein coding genes, rRNA genes, tRNA genes, whole mtDNA, and all mitochondrial and nuclear data merged). Strikingly, all topologies have almost identical likelihood values, resulting in largely non significant likelihood tests. *Elephas-Mammuthus* could be rejected only for the tRNA data partition (at p-value < 0.05 under an Approximatively Unbiased test; Table 2). Interestingly none of the tests performed on complete mitochondrial genome data sets was able to corroborate Rogaev et al. (2006) reports of significant support for the *Mammuthus-Elephas* clade. Noteworthy, Rogaev et al. (2006) did not provide any details on how likelihood ratio tests were performed to discriminate between alternative topologies (while this procedure is still unknown to most phylogeneticists; see Felsenstein, 2004 for a discussion of the inadequacy of likelihood ratio in testing alternative topologies).

We then decided to count the total number of synapomorphies of the three possible pairs of taxa, using the state of Hyrax and Dugong sequences to polarize character changes (Table 2). Merging all data, none of the three pairs exhibits significant deviation from the mean number of synapomorphies (Chi-square, p-value = 0.774), suggesting similar parsimony support for the three alternatives. All but ribosomal RNA (p = 0.004) partitions of the data yielded non-significant deviations as well (0.158 < p-value < 0.819). Such a pattern might be indicative either of lineage-sorting effects among mammoths (one gene leading to a first phylogenetic signature whereas another one to the opposite), of differential parallel or convergent evolution in some genes, or of poor polarization of characters. Indeed, Hyrax and Dugong have diverged from the elephant lineage about 65 MYA.

**Figure 1.**
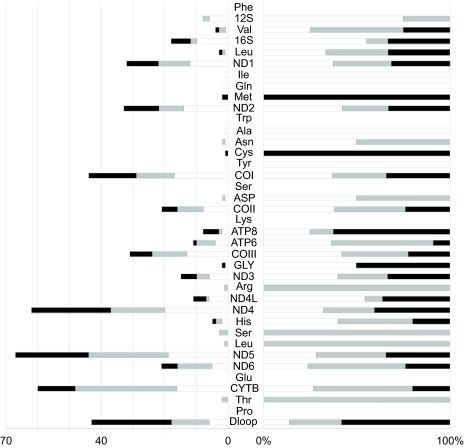
Absolute (Left) and relative (Right) number of synapomorphies for the ((E,L),M) (Black), ((M,L),E) (Grey) and ((M,E),L) (White) topologies. Each bar represents a given gene in the dataset.

In theory, Bayesian analyses reported in Rogaev et al. (2006) could support the lineage-sorting hypothesis (Table 1) since different topologies were supported by different genes. Since mitochondria are mostly non-recombinant in animal (e.g. Barr, Neiman and Taylor, 2005), this explanation is unlikely. Furthermore, while the *Mammuthus-Elephas* relationship was supported by both partitioned Bayesian analyses (p = 0.931 and 0.751 for P46 and P10 respectively), the *Mammuthus-Loxodonta* and *Elephas-Loxodonta* (P10 only) alternatives were both present in the 95% credibility interval. Similarly, none of the three possible pairs of taxa exhibits significant differences in the number of synapomorphies (Figure 1). Moreover, whatever the framework or alternatives, hidden branch support is systematically detected in our data set. Such results are rather unexpected since alternatives are mutually incompatible. One possible explanation for the strong and incompatible Bayesian posterior probabilities reported by Rogaev et al. (2006) would imply a hard polytomy between *Mammuthus-Loxodonta* and *Elephas*. Lewis and co-workers (2005) have indeed convincingly demonstrated that standard MCMC procedure tends to become unpredictable, including strong shift from one to another alternative, when the true phylogeny is a hard or near hard polytomy (but see Kolaczkowski and Thornton, 2006). Alternatively, overall results could be due to inadequate polarization of the data. This finding is corroborated in *littera* since different rooting procedures (or phylogenetic methods) often come to opposite conclusions (Table 1).

In that context, one possibility would be to build unrooted trees and check the stability of molecular clock along the branches, to infer the most probable midpoint position of the root. This strategy has already been followed in several studies (Table 1) but leaves no possibility to test if the resulting topology is better than the alternatives. We thus consider tree rooting as a prerequisite before drawing any definite conclusion with regards to the phylogenetic relationships of mammoths and elephants and possible hard polytomy. The American mastodon (*Mammut americanum*) lineage would be very useful for that purpose since it diverged from the lineage of mammoth and elephants about 24 MYA, that is 40 MY later than the Hyracoidea (e.g. Hyrax)—Sirenia (e.g. Dugong) —Proboscidia (elephantids) split (Shoshani and Tassy, 1996). Moreover, the American mastodon (*Mammut americanum*) became extinct at the Late Glacial Maximum, and specimens from that time range are compatible with ancient DNA recovery (Gilbert et al. 2005). Such a mastodon DNA sequence (circa 10 KYA) was used in Yang et al. (1996), but several authors have raised concerns on the authenticity of the mammoth sequences recovered (Thomas et al. 2000; Debruyne et al. 2003).

Finally, we should bear in mind that star trees are the null hypothesis in phylogenetic reconstructions. Thus if the adaptive radiation among elephantids occurred very rapidly (ca. 500 KY) as recently suggested (Krause et al. 2006), this near hard polytomy might require extensive data to be solved. One fruitful strategy would most probably be to take advantage of the ongoing *Loxodonta* genome project and of the 13 Mbp of the mammoth genome already published (Poinar et al. 2006). *In silico* analyses might identify most-informative candidate genes and enable to design primers in order to collect orthologous sequences in both *Elephas* and mastodons. New technological advances in two-round multiplex-PCR (Krause et al. 2006; Römpler et al. 2006) and large-scale sequencing (Poinar et al. 2006), combined with the exceptionally-well preserved mammoth specimens from permafrost, make this an exceptional model for the genomic study of speciation.

## Figures and Tables

**Table 1.** Molecular support for unravelling intra-Elephantidae phylogenetic relationships.

**Reference**	**Gene**	**Length (nt)**	**Sites**	**Topology^a^**	**Type of analysis**	**Bootstrap (%) / Posterior Probability**	**Root^a^**
Hagelberg et al. 1994	Cyt b	278	all	(M,L),E	Distance	-	-
Höss et al. 1994	16S rDNA	93	all	*uninf.*	Distance	-	-
Yang et al. 1996	Cyt b	228	all	(M,E),L	MP	74	Ma
Ozawa et al. 1997	Cyt b	670	1+2	(M,E),L	NJ, MP	72, 72	Dd, Hg, Tm, Pc
	Cyt b	330	aa	(M,E),L	NJ, MP	90, 91	Dd, Hg, Tm, Pc
Noro et al. 1998	Cyt b	1137	all^b^	(M,L),E	NJ, MP	92, 73	Dd, Db, Eg, Bt
	12S rDNA	961	all	(M,L),E	NJ, MP	55, 81	Dd, Tm, Pc, Ddo, Db, Eg, Bt
Greenwood et al. 1999	Cyt b	305	all	(M,L),E	Distance	-	-
	16S rDNA	94	all	(M,E),L	Distance	-	-
	28S rDNA	138	all	(M,E),L	Distance	-	-
	IRBP	43	all	(M,E),L	Distance	-	-
	A2AB	57	all	*uninf.*	Distance	-	-
	vWF	114	all	*uninf.*	Distance	-	-
Thomas et al. 2000	Cyt b	255	all	(M,L),E	MP, ML	84, 67^c^	Dd
	Cyt b	453	all	(M,L),E	ML, BI	67, 0.43^d^	Midpoint rooting
Debruyne et al. 2003	Cyt b	228^c^	all	(M,L),E	NJ, MP	<50, 25	Ma
	Cyt b	561	all	(M,L),E	NJ, MP	63, 88	Dd, Hg, Tm, Pc
	Cyt b	561	all	(M,E),L	ML	not provided^d^	Dd, Hg, Tm, Pc
Capelli et al. 2006	5 nuclears	701	all	(E,L),M	NJ, MP, ML	70, 61, 77^d^	Pc
	5 nuclears	677	all	(M,E),L	NJ, MP, ML	100, 100, 100	Midpoint rooting
Krause et al. 2006	mt genome	16770	all	(M,L),E	NJ, ML, BI	73, 56, 0.97	Dd
	mt genome	16770	all	(M,E),L	MP	62	Dd
	mt genome	16770	all	(M,E),L	NJ, MP, ML, BI	83, 93, 79, 0.91	Pc
	mt genome	16770	all	(M,E),L	NJ, MP, BI	87, 90, 1.0	Dd, Pc
	mt genome	16770	all	(M,L),E	ML	54	Dd, Pc
	mt genome	16770	all	(M,E),L	ML, BI^e^	97, 0.998	Midpoint rooting
Rogaev et al. 2006	mt genome	16842	all	(M,E),L^f^	MP, ML, BI	95, 8, 0.88–1.0^g^	Dd, Pc
	12S rDNA	962	all	(M,E),L	BI	0.72	Dd, Pc
	ATP6	669	all	(M,E),L	BI	0.68	Dd, Pc
	COX1	1551	all	(M,E),L	BI	0.90	Dd, Pc
	COX3	784	all	(M,E),L	BI	0.92	Dd, Pc
	Cyt b	1137	all	(M,E),L	BI	0.76	Dd, Pc
	ND1	957	all	(M,E),L	BI	0.85	Dd, Pc
	ND4L	297	all	(M,E),L	BI	0.99	Dd, Pc
	ND6	528	all	(M,E),L	BI	0.96	Dd, Pc
	COX2	684	all	(M,L),E	BI	0.56	Dd, Pc
	ND3	346	all	(M,L),E	BI	0.92	Dd, Pc
	ND5	1812	all	(M,L),E	BI	0.89	Dd, Pc
	16S rDNA	1566	all	(E,L),M	BI	0.88	Dd, Pc
	ND2	1044	all	(E,L),M	BI	0.94	Dd, Pc
	ND4	1368	all	(E,L),M	BI	1.0	Dd, Pc
	ATP8	201	all	*uninf.*	BI	-	Dd, Pc

^a^ M: *Mammuthus primigenius;* E: *Elephas maximus*; L *Loxodonta africana*; Ma: *Mammut americanum*; Dd: *Dugong dugon*; Tm: *Trichechus manatus*; Pc: *Procavia capensis*; Hg: *Hydrodamalis gigas*; Db: *Diceros bicornis*; Eg: *Equus grevyi*; Bt: *Bos taurus*; Ddo: *Dendrohyrax dorsalis*.

^b^ but (M,E),L when considering only transversions or translated sequences

^c^ casts doubt on the 228-nt sequence reported in Yang et al. 1996

^d^ support for this topology but similar likelihood / probability for alternative topologies

^e^ trifurcation rejected based on parsimony statistics

^f^ significant ML ratio test

^g^ two different models were used for BI inference

**Table 2.** Likelihood values and number of substitutions in favor of each topology.

	**Gene**	**Length (nt)**	**Sites**	**(M,E),L**	**-Ln L**	**(E,L),M**	**Number of synapomorphies**		
					**(M,L),E**		**(M,E)**	**(M,L)**	**(E,L)**
All	mtDNA+nucDNA (n = 48)	17917	all	56081.75	56081.19	56082.85	181	145	153
	mtDNA (n = 38)	17072	all	54272.15	54272.09	54273.97	176	144	150
	nucDNA (n = 10)	845	all	1715.07	1715.48	1714.53	5	1	3
Proteic	mtDNA+nucDNA (n = 18)	11699	all	38164.79	38165.56	38166.03	144	121	127
	mtDNA (n = 13)	11396	all	37446.06	37447.52	37447.99	141	121	126
	nucDNA (n = 5)	303	all	623.53	623.57	623.57	3	0	1
	mtDNA+nucDNA (n = 18)	7821	1+2	19861.75	19859.50	19857.60	32	30	38
	mtDNA (n = 13)	7619	1+2	19490.58	19488.61	19486.74	31	30	38
	nucDNA (n = 5)	202	1+2	346.94	346.94	346.94	1	0	0
	mtDNA+nucDNA (n = 18)	3920	2	8102.64	8104.73	8104.96	9	6	5
	mtDNA (n = 13)	3819	2	7928.22	7930.31	7930.51	8	6	5
	nucDNA (n = 5)	101	2	163.90	163.90	163.90	1	0	0
Ribosomal	mtDNA (n = 2)	2526	all	6613.81	6613.42	6613.77	17^k^	4^k^	6^k^
tRNA	mtDNA (n = 22)	1522	all	4112.11	4110.83	4114.72^a^	11	11	5
Non coding	mtDNA+nucDNA	2224	all	6861.46	6861.46	6859.50	9	9	15
	mtDNA	1682	all	5736.27	5736.27	5734.48	7	8	13
	nucDNA	542	all	1073.18	1074.23	1072.65	2	1	2

^a^ indicates topologies that are significantly worst than the most likely alternative based on AU test.

^k^ indicates a significantly different number of synapomorphy. Mp, Em, La, Dd, Pc stand respectively for Mammoth, Asian and African elephants, Dugong and Hyrax accession numbers: Complete mtDNA = DQ316067(Mp) DQ188829(Mp) DQ316068(Em) LAAJ4821(La) DQ316069(La) AY075116(Dd) DDU421723(Dd) AB096865(Pc); BGN 5’ = DQ267154(Mp) DQ265809(Em) DQ265820(La) DQ265813(Pc); BGN 3’ = DQ265811(Mp) DQ265809(Em) DQ265820(La) DQ265813(Pc); CHRNA1 5’ = DQ267155(Mp) DQ265827(Em) DQ265838(La) DQ265831(Pc); CHRNA1 3’ = DQ267156(Mp) DQ265827(Em) DQ265838(La); GBA = DQ265846(Mp) DQ265844(Em) DQ265843(La) DQ265848(Pc); LEPR = DQ265868(Mp) DQ265866(Em) DQ265888(La) DQ265871(Pc); VWF 5’= AF154875(Mp) DQ265898(Em) DQ265919(La) DDU31608(Dd) DQ265902(Pc); VWF 3’ = consensus of AF154873/AF154874(Mp) DQ265898(Em) DQ265919(La) DDU31608(Dd) DQ265902(Pc); IRBP = AF155042(Mp) AY243443(Em) LAU48711(La) DDU48583(Dd) PCU48586(Pc); A2AB = AF154876(Mp), Y12525, (Em), AF154877(La), Y15947(Dd), Y12523(Pc).

**Table 3:** Hidden Branch Support.

	**Parsimony**			**Likelihood**		
	**(M,E),L**	**(M,L),E**	**(E,L),M**	**(M,E),L**	**(M,L),E**	**(E,L),M**
∑BS_ind_	2	–45	–40	–10,92	–12,05	–15,64
BS	28	–32	–28	–0,56	0,56	–1,65
HBS	26	13	12	10,37	12,6	13,98

∑BS_ind_ : sum of branch support (BS) scores for that node from each data partition. BS : difference in the number of character steps (or likelihood difference) between the best topology with and without that node. HBS (Hidden branch support) = BS-∑BS_ind_. For further details see Gatesy et al. 1999.
